# Enhancing academic performance and student engagement in health education: insights from Work Station Learning Activities (WSLA)

**DOI:** 10.1186/s12909-024-05478-z

**Published:** 2024-05-03

**Authors:** Judit Sánchez, Marta Lesmes, Margarita Rubio, Beatriz Gal, Antonio S. Tutor

**Affiliations:** 1 Departamento de Educación e Innovación Educativa, Universidad Europea de Madrid, Madrid, Spain; 2 Departamento de Medicina, Facultad de Ciencias Biomédicas y de la Salud, Universidad Europea de Madrid, Madrid, Spain; 3 Departamento de Ciencias Médicas Básicas, Facultad de Medicina, Universidad San Pablo-CEU, CEU Universities, Urb. Montepríncipe s/n, Madrid, 28668 Spain

**Keywords:** Active learning methodologies, Integrated curriculum, Basic and clinical sciences

## Abstract

Making health science students aware of the importance of basic science knowledge for professional practice is a major educational challenge, especially during the early years of preclinical courses. Here, using an integrated curricular approach, we analyze whether Work Station Learning Activities (WSLA), which combine active learning methodologies for teaching basic science in clinical scenarios, can help to develop deeper learning and student engagement. In order to increase student motivation, we evaluated the effectiveness of WSLA using statistical analyses and an observation tool based on the ICAP (Interactive, Constructive, Active, and Passive) framework, which categorizes learning tasks based on the nature of student engagement. Statistical analyses revealed positive correlations between the different summative evaluations along the development of the activities, indicating the learning process inherent to WSLA progression and affirming the positive influence of WSLA on academic outcomes. Comparing the pre- and post-tests, students scored significantly higher on the post-test (statistically significant *p* < 0.001). WSLA promotes both constructivist and interactive learning, as validated by its alignment with the ICAP model. The study examines student engagement through systematic observation, revealing a relationship between student engagement and final grades. Students who exhibit constructive learning consistently earn higher grades, emphasizing the positive impact of active engagement. Thus, passive behavior profiles show a significant proportion of fails (40%), while constructive profiles stand out as the sole recipients of the coveted excellent rating. Ultimately, this study contributes to our understanding of the effectiveness of WSLA in promoting active learning and enhancing student engagement within integrated health education curricula. It highlights the importance of active learning behaviors for academic success and suggests avenues for further research to optimize integrated teaching methodologies in medical education.

**Supplementary Information:**

The online version contains supplementary material available at 10.1186/s12909-024-05478-z.

## Background

A common challenge faced by basic science educators teaching undergraduate health-related courses is students’ limited recognition of the practical applications of basic science concepts typically addressed in early years. Integrating basic and clinical sciences can help students to make this connection easier. In fact, integration has become mainstream in reputed programs [1, 2]. Finnerty [3] revisited the conventional perspective, embracing an integrated “basics to clinics” approach, and proposed the infusion of fundamental sciences into clinical studies. At the same time, a clinical perspective should be included from the initial formative years to better equipe students with a deeper understanding of basic sciences application to clinic. It has been described that active methodologies are better suited for the development of such an integrated curricula [4]. Active methodologies are defined by educational researchers as any activity that ‘involves students in doing things and thinking about the things they are doing’ by engaging them cognitively and meaningfully with the materials [5], leading to a better understanding of complex ideas and mastering difficult skills [6]. All this suggest that students would be better able to handle with difficulties if they actively engage in the cognitive processes required to build connections amongst separate information pieces [7]. Recognizing the value of an integrated curriculum in promoting a holistic understanding of clinical and basic sciences is thus critical [3]. Current trends in health education emphasize an integrated approach that uses clinical cases as a central guiding framework to actively engage students in their learning process. This approach aims to develop critical thinking, clinical reasoning, and a conducive, low-stress learning environment [4, 8]. The shift toward integrated curricula involves a sequential transition from independent subjects to cohesive, integrated programs [9–11]. The Harden Integration Ladder [12] serves as a valuable tool for planning and evaluating medical education curricula, outlining 11 steps that measure the level of integration between discipline-based and instruction.

According to Harden’s model, our educational approach aligns with the correlation level, which implies a curriculum structured around individual disciplines with an integrated teaching approach incorporated alongside subject-based instruction [12]. To achieve this, we introduced Work Station Learning Activities (WSLA) as a flexible and scalable teaching tool for progressive integration of basic science subjects into the biomedical curriculum [13]. WSLA aims to bridge concepts across different subjects, promote active learning in a team-based environment, and provide pedagogical advantages for progressing towards a fully integrated curriculum.

**Fig. 1:**
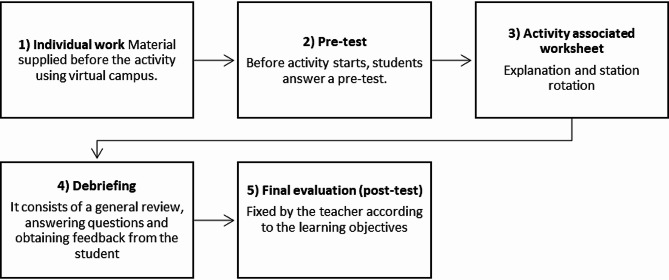
The different steps of WSLA methodology

The WSLA methodology allows to teach a particular topic (e.g. pH regulation) across a series of different workstations run in a clinical scenario. The steps (Fig. 1) involved in WSLA include: (1) providing relevant content on virtual campus prior to the activity to ensure student readiness, (2) a pre-test to measure student preparation, (3) presentation of a clinical scenario with workstations followed by team-based activities (worksheets), (4) a general review, and assessment based on learning objectives, all followed by (5) a post-test.

Previous studies have identified WSLA as an effective strategy to promote deep learning and improve academic performance in medical early years curricula [14, 15]. Qualitative analysis suggests that students perceive WSLA as a motivating and constructive framework for understanding complex concepts, highlighting the role of both professors and students’ in learning, and their ability to work in groups [14].

Moreover, to gain further insights on the pedagogical model, we evaluated WSLA approach within the ICAP framework and found strong alignment [16]. The ICAP framework, introduced by Chi and Wylie [7], categorizes learning tasks based on the nature of student engagement. It identifies four types of engagement: interactive (involves active participation of students in discussions and collaboration with other students or the facilitator), constructive (refers to the generation of new ideas or the application of learned concepts to specific situations), active (characterized by direct manipulation of materials or the performance of practical activities), and passive (focuses on observation without active participation in the activity). It suggests that as students become increasingly engaged with learning materials, their learning outcomes improve [7, 17]. However, we can still deepen our understanding of how students themselves perceive WSLA. Understanding students’ experiences can guide future development of WSLA and its alignment with the ICAP model.

In the context described, we propose that students engaged in Work Station Learning Activities (WSLA) will demonstrate increased levels of engagement and participation compared to those in traditional classroom settings. We anticipate that these increased levels of engagement will be consistent with the behavioral profiles outlined in the ICAP model and will positively correlate with academic achievement. Furthermore, we hypothesize that the immersive nature of WSLA will foster a deeper understanding of basic science concepts, resulting in improved post-test scores compared to pre-test scores within the same cohort. In addition, we hypothesize that the collaborative structure inherent in WSLA will foster teamwork skills among students, thereby enhancing their ability to collaborate, which we anticipate will be reflected in a positive correlation between teamwork scores and post-test performance.

Therefore, this study aims to analyze the positioning of WSLA within a constructive learning framework, and to delve into the WSLA methodology and its relationship to ICAP. Evaluation measures include pre-test, teamwork (activity-related worksheets), and post-test grades, as well as systematic classroom observations to assess the nature and degree of student interaction.

## Methods

### Cohort description

This study was conducted amongst first-year medical students at the Universidad Europea de Madrid, Spain, during the academic years 2019–2020 (268 students) and 2020–2021 (233 students). The study was approved by the Ethics Committee Universidad Europea de Madrid (CIPI/20/092). Students provided their written informed consent to participate in this study.

Three WSLA sessions were designed, each integrating different basic science courses: session 1 focused on a clinical case of pH regulation (Biochemistry and Physiology integration), session 2 on a clinical case of carpal tunnel syndrome (Anatomy and Physiology integration), and session 3 on a clinical case of radiculopathy (Anatomy and Physiology integration). Each hands-on session lasted 2 h.

### Quantitative study

To accomplish our study objectives, a quantitative study was conducted to examine inherent learning during the WSLA and its correlation with student engagement. In each session, students had to complete a pre-test, a post-test and a team-based activities (worksheet). Each test consists of ten multiple-choice questions. Correctly answered question scores one point, incorrectly answered question has a penalty of -0.33 points. The discrimination index and the level of difficulty of the questions have been calculated to ensure the homogeneity of the tests. Regarding the team-based activities (worksheets), students’ progress through the different stations, each presenting integrated content activities that they must solve as a group. These activities include analyzing diagrams, drawings and images, making hypotheses, and reasoning about the content, as well as identifying structures and components, among other tasks that require reasoning and justification.

To assess academic performance, data were collected from the 2019–2020 and 2020–2021 cohorts. Statistical analyses were conducted to compare grades obtained on pre-tests, post-tests, team-based activities (worksheets), and final grades (50% pretest grade + 25% teamwork grade and 25% post-test grade). Marks are from 0 to 10. Student’s achievement was defined as “fail” (< 5 mark), “pass” (5-6.9), “distinguished” (7-8.9) and “excellent” (9–10).

Statistical analyses included nonparametric Spearman’s rank correlation to examine correlations between different grades for both cohorts. A comprehensive analysis of three WSLA sessions from both cohorts was also conducted using the Wilcoxon signed-rank test to assess improvement in post-test scores. An independent analysis of activities within these sessions was also conducted using a Friedman test with Bonferroni correction to compare pre-test scores.

### Analysis of student engagement

An observational instrument was developed based on student behaviors as categorized by the ICAP framework (see Annex 1). The observable behaviors during the activities defined the student´s engagement as interactive, constructive, active, and passive modes. The instrument was developed (during 2019–2020 academic year) and refined after a preliminary evaluation (during the first WSLA of 2020–2021 academic year) and was used to systematically observe students during the second and third WSLA sessions of 2020–2021.

**Fig. 2:**
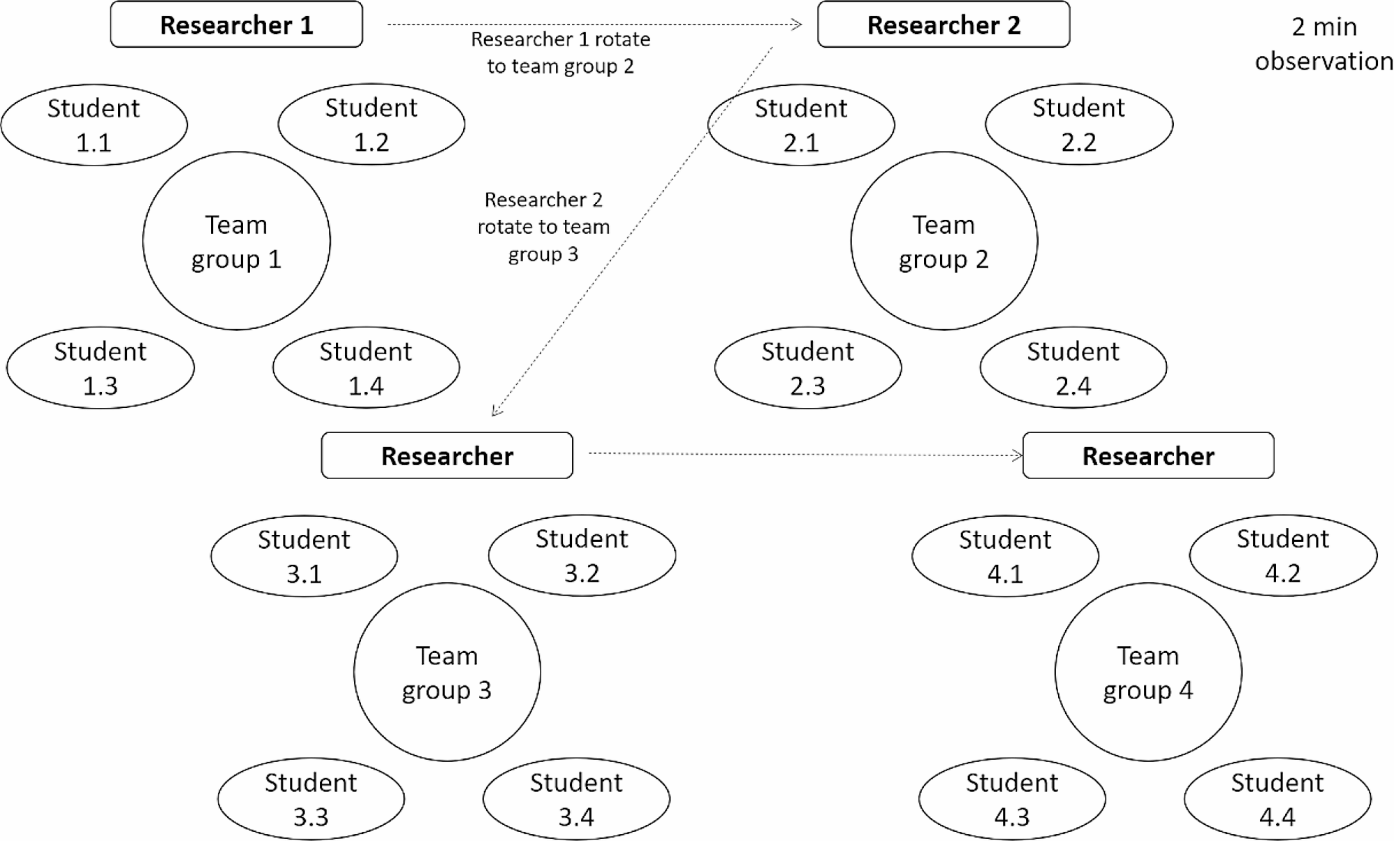
Data collection

A systematic data collection process involved assigning numerical identifiers to team groups and individual students (Fig. 2). Two researchers simultaneously observed student groups at 2-minute intervals, marking appropriate categories based on perceived engagement behaviors. The observers rotated until all groups were observed multiple times during the WSLA session. Students were categorized based on the predominant type of behavior, while developing the activity. We defined an internal criterion that was 75% of behaviors to define a specific engagement excluding cases of mixed behaviors. Observational data were obtained for 261 behaviors out of 466 possible cases, but only 156 could be classified into a well-defined ICAP profile cohort to analyze student engagement during WSLA sessions.

Each student was categorized based on the mode (type of behavior) for which they received the most marks. We only considered where the type of behavior was consistently clear, for example, if a student had 10 marks in passive mode and only 2 marks in other kinds of behaviors, we considered that student’s engagement as passive because, the student’s behavior was predominantly passive. If a student exhibited conflicting behavior, they were excluded from consideration. For example, if a student had 3 marks in passive mode and 3 marks in active mode, we didn’t consider this student because we required a distinct and observable engagement behavior for most of the session.

This methodological approach was designed to provide a comprehensive understanding of both the inherent learning outcomes and student engagement during WSLA sessions, shedding light on the effectiveness and dynamics of this teaching methodology in medical education.

A statistical analysis using the chi-squared test was used to investigate the potential relationship between student engagement and his or her final grade. The purpose of this analysis was to determine whether observable student behaviors were associated with specific final grade categories, namely, fail, pass, distinguished, or excellent. In essence, the chi-squared test sought to determine whether certain student profiles were associated with a tendency to achieve certain final grade outcomes.

In addition, the Kruskal-Wallis statistical test was used to evaluate the performance of student profiles on various assessments, including pre-tests, post-tests, activity-related worksheets, and final grades. This analysis sought to determine whether student behavior played a discriminative role in influencing their level of performance on each category of assessment. In essence, the Kruskal-Wallis test was used to determine if there were significant differences in academic performance across different student profiles within each assessment.

For all statistical analyses the threshold for statistical significance was set at 0.05.

## Results

### Assessment of inherent and specific academic performance in WSLA sessions

To assess intrinsic and specific academic performance in the context of WSLA sessions, a comprehensive study was conducted. This evaluation included grades derived from various assessments, including pre-tests, team-based activities (worksheets), post-tests, and final grades. The primary goal of this analysis was to identify correlations between these assessment components across, the 2019–2020 (*n* = 268 students) and 2020–2021 (*n* = 233 students) cohorts.

As shown in Table 1, Spearman’s rank analysis revealed a significantly positive correlation, indicating that an increase in one set of grades corresponds to an increase in the other. By stressing the significance levels (*p* < 0.01), we identified the most robust correlations observed in our data, with consistent trends across both cohorts.

Upon closer examination of Table 1, the most robust correlation between pre-test scores and final grades is observed in both the 2020–2021 cohort (correlation coefficient = 0.923, p-value < 0.01) and the 2019–2020 cohort (correlation coefficient = 0.875, p-value < 0.01). This reinforces the notion that higher pre-test scores correlate with higher final grades. Another significant correlation is evident when comparing post-test scores to final grades in both cohorts (correlation coefficient = 0.668, p-value < 0.01 for 2020–2021, and correlation coefficient = 0.592, p-value < 0.01 for 2019–2020), suggesting that superior post-test performance is associated with higher final grades.

In addition, the third most significant correlation for these cohorts manifests itself in different ways while maintaining remarkably similar data trends. In the 2020–2021 cohort, there was a notable correlation between pre-test and post-test scores (correlation coefficient = 0.408, p-value < 0.01), highlighting that students with higher pre-test scores generally perform better on post-tests. Conversely, for the 2019–2020 cohort, the third strongest correlation is associated with teamwork and final grades, characterized by a correlation coefficient of 0.333.

**Table 1:** Correlations in academic performance (Spearman’s rank correlation)

	2019–2020 academic year (*n* = 268)	2020–2021 academic year (*n* = 233)
**Correlation between pre-test and teamwork**		
Correlation coefficient	0.108^**^	0.080^*^
Sig. (bilateral), p-value	0.002	0.035
**Correlation between pre-test and post-test**		
Correlation coefficient	0.263^**^	0.408^**^
Sig. (bilateral), p-value	< 0.001	< 0.001
**Correlation between teamwork and post-test**		
Correlation coefficient	0.148^**^	0.101^**^
Sig. (bilateral), p-value	< 0.001	0.008
**Correlation between pre-test and final grades**		
Correlation coefficient	0.875^**^	0.923^**^
Sig. (bilateral), p-value	< 0.001	< 0.001
**Correlation between post-test and final grades**		
Correlation coefficient	0.592^**^	0.668^**^
Sig. (bilateral), p-value	< 0.001	< 0.001
**Correlation between teamwork and final grades**		
Correlation coefficient	0.333^**^	0.271^**^
Sig. (bilateral), p-value	< 0.001	< 0.001

*. The correlation is significant at the 0.05 (bilateral)

**. The correlation is significant at the 0.01 level (bilateral)

**Fig. 3:**
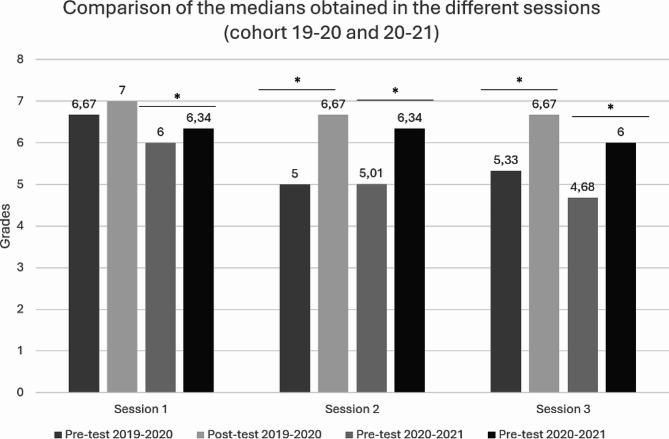
Comparison between pre-test and post-test scores across various WSLA sessions. Median values were utilized in the construction of this graph.* *p* < 0.001

Following the completion of each of the three sessions, a comprehensive analysis was conducted to compare the grades achieved. Figure 3 shows the median pre-test and post-test scores for the 2019–2020 and 2020–2021 cohorts.

A paired sample Wilcoxon signed-rank test was used to determine the presence of statistically significant differences in the scores obtained. The results, shown in Fig. 3, revealed significant differences (*p* < 0.001) between the pretest and posttest in all the WSLA and academic years analyzed.

Following an independent analysis of the activities, a study we aimed to determine whether students exhibited an increased commitment to preparing for successive WSLA sessions. It was hypothesized that increased pretest scores in each session would indicate increased readiness for subsequent sessions. Significantly, scores showed a marked and statistically significant decline (*p* < 0.01) as the WSLA sessions progressed, with scores of 6.67, 5.00, and 5.33 for sessions 1, 2, and 3 in the 2019–2020 academic year and 6, 5.01, and 4.68 for the 2020–2021 academic year.

### Student’s engagement

In the context of student engagement, our focus centered on evaluating the nature and extent of student interaction and group work activities during WSLA sessions through systematic classroom observation. The findings revealed significant differences (*p* = 0.012), suggesting a significant relationship between students’ engagement and their final grades.

**Fig. 4:**
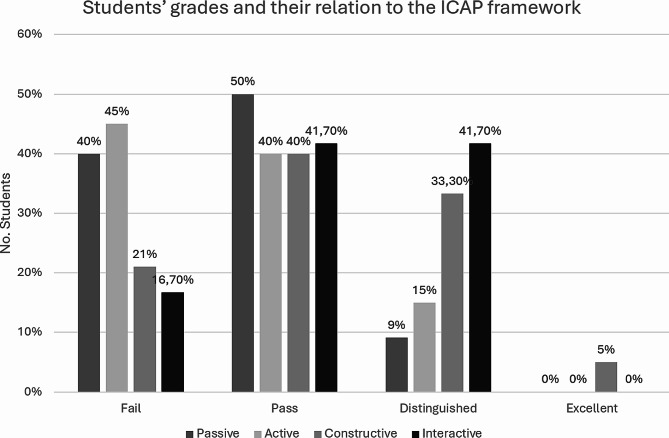
Student’s grades percentage related with their behaviors

Analysis of Fig. 4 unveiled distinctive academic outcomes associated with different student profiles. Among those with a passive profile, 40% achieved a “fail”, while 50% achieved a “pass.” Interestingly, the remaining 9.1% attained a “distinguished”, with no students in this category receiving an “excellent”. Conversely, students with an active profile exhibited a different distribution: 45% achieved a “fail”, 40% achieved a “pass,” and 15% attained an “distinguish”. Notably, no students in this category obtained an “excellent”.

Turning attention to students categorized with a constructive profile, 21% received a “fail,” while 40% fell within the “pass” range. Remarkably, 33.30% achieved a “distinguished”, and 5% received the coveted “excellent” rating, distinguishing this behavioral profile as the sole recipient of the highest grade. Lastly, among students classified as interactive, only 16.70% received a “fail”, while 41.70% achieved a “pass,” mirroring the distribution observed in those with a constructive profile. The most notable distinction associated with interactive behavior was the attainment of a “distinguished” by 41.70%, representing the highest proportion of students securing this designation within the dataset.

**Fig. 5:**
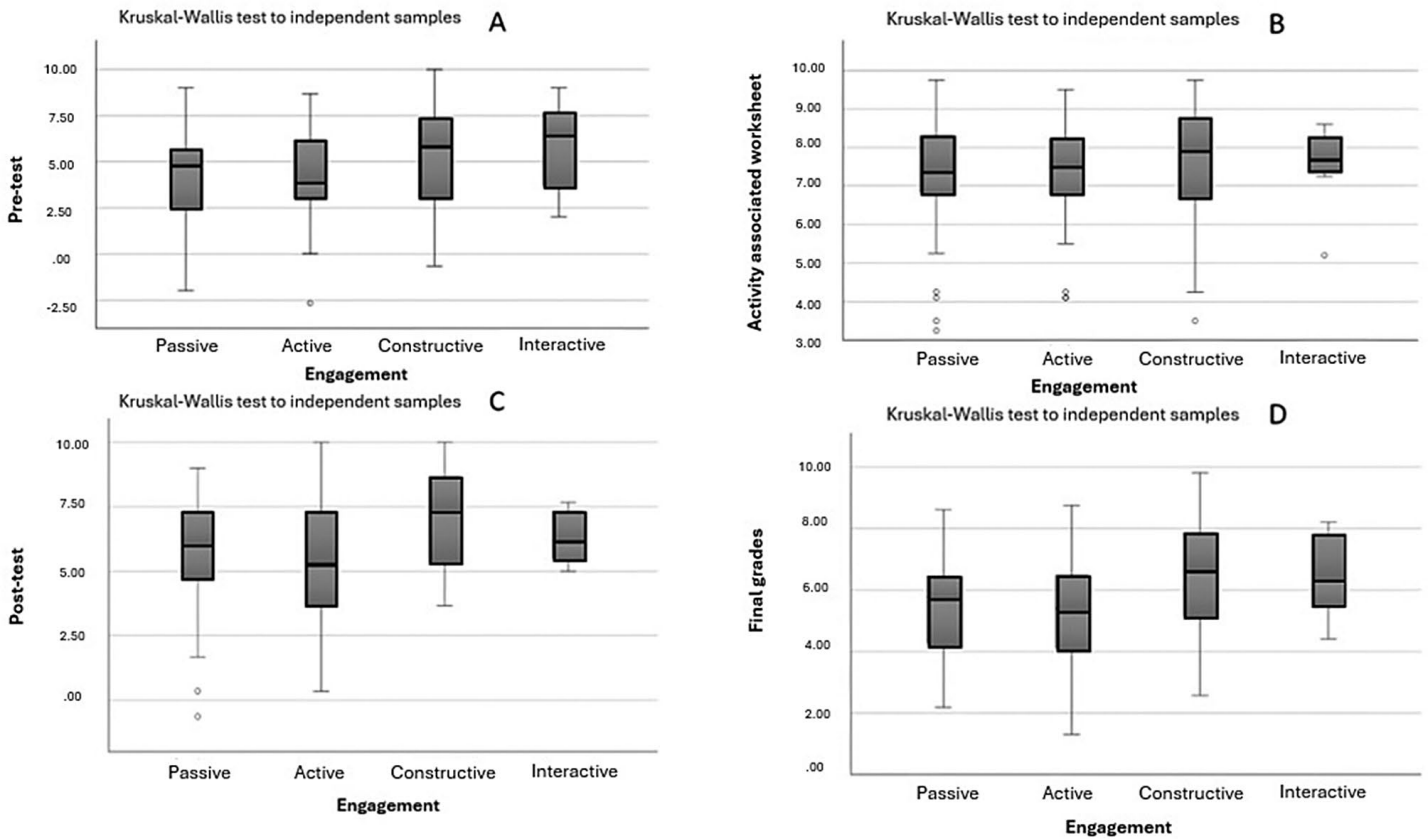
The observation of differences in the pre-test (**A**), team-based activities (worksheets) (**B**), the post-test (**C**) and the final grades (**D**), compared to the student´s engagement

Finally, with regard to student engagement, the Kruskal-Wallis test with Bonferroni correction was used to examine potential differences in WSLA different steps student grades based on behavior. As shown in Fig. 5, both the pretest (A) and the teamwork assessment (B) showed no significant differences in grades based on student engagement (adjusted p-value > 0.05). However, when the results of the post-test (C) and the final grade (D) are compared with the students’ engagement, the graph shows that students who demonstrated constructivist engagement received higher grades than those who demonstrated active, passive, or interactive engagement (adjusted p-value ≤ 0.01).

## Discussion

Educators, especially in health-related undergraduate programs, often face the challenge of students struggling to see the practical applications of basic science concepts in their future careers. Our previous research addresses this common problem by introducing the WSLA methodology within an integrated curriculum framework that aligns with the correlation level of Harden’s Integration Ladder [12]. This model aims to promote a holistic understanding of clinical and basic sciences, encourage critical thinking and clinical reasoning, in the context of a low-stress, conducive learning environment [13, 14].

The results of this current study continue to delve into the effectiveness of the WSLA methodology in the context of undergraduate health-related courses. Statistical analyses revealed notable correlations and findings that contribute to our understanding of the impact of WSLA on student performance.

We found a robust correlation between pre-test scores and final grades for both 2019–2020 and 2020–2021 cohorts. This underscores that higher pre-test scores are associated with higher final grades, highlighting the importance of thorough preparation prior to attending WSLA sessions, as noted in previous research [18, 19]. A solid understanding of the theoretical framework allows students to approach the practical aspect with a deeper understanding of the scientific principles involved. This not only enhances the learning experience, but also promotes a deeper understanding of the subject matter [20].

The positive impact of WSLA extends to post-test scores, indicating that students have a more comprehensive understanding of content tested after the WSLA session. In addition, the increase in grades suggests that an active learning intervention facilitated deep learning and student engagement. Interactive and participatory teaching methods tend to foster a deeper connection with the subject matter, encouraging students to invest more effort and attention in their studies [19–21].This active involvement enhances their learning experience and has a positive impact on academic performance. In addition, the observed improvement in grades may indicate the development of critical thinking skills and deeper conceptual understanding amongst students. A well-designed instructional methodology encourages students to analyze, synthesize, and apply knowledge beyond rote memorization. This higher-order thinking skills contribute not only to improved test scores, but also to deeper mastery of subject matter [20–22].

The interactive and constructive elements inherent in WSLA that promote active engagement and participatory learning contribute significantly to this positive outcome, which is consistent with broader research supporting the effectiveness of active learning methods, including team-based learning (TBL) [7, 22–24]. Furthermore, the study highlights the promotion of constructive engagement in the learning process, with activities such as concept mapping, which fall under the constructive mode of the ICAP framework and prove more effective than passive or active modes, which is consistent with the existing literature on constructive learning approaches, such as problem-based learning (PBL) implementations [17, 25, 26].

We found a positive correlation between student engagement behaviors during WSLA sessions and final grades which adds weight to the idea that constructive learners consistently achieve higher grades on both post-tests and final evaluations. This reinforces the concept that active participation and constructive engagement contribute to a more positive overall learning experience [9, 27].

Importantly, our study also draws attention to the challenges of maintaining student engagement over time, as evidenced by the decline in pre-test scores across successive WSLA sessions. This finding underscores the inconsistency in achieving improved scores as the course progresses through its sessions. It is of utmost importance that educators proactively emphasize to students the critical importance of thorough preparation before engaging WSLA and similar types of methodologies that do require of active student participation. This deliberate effort is essential not only to maximize the benefits of the activities, but also to consolidate the content addressed throughout the learning process. Factors such as increased academic workload or burnout may contribute to this decline, highlighting the need to address these challenges to maintain the effectiveness of the WSLA throughout the course [28–29].

A possible limitation of the WSLA is the additional workload associated with the preparation phases. In fact, as mentioned above, contrary to what we expected, we observed that the pre-test grade, which is related to the autonomous work that the student must develop prior to performing the activity, gradually decreased as the course progressed. We can relate this fact to the increase in the number of assignments that students experience throughout the academic year. This aspect has been highlighted in the literature in relation to the additional workload associated with active methods [30]. One possible solution would be to strategically integrate different active methodologies across the curriculum to mitigate workload differences and avoid overloading. Exploring this possibility is a promising direction for improvement.

In conclusion, our study provides valuable additional insight on the effectiveness of WSLA within an integrated curriculum, highlighting its positive impact on learning outcomes and student engagement. The correlations identified between assessment components and the influence of engagement on academic performance underscore the potential of WSLA as a valuable pedagogical tool in health-related education. It highlights the importance of early preparation and active learning behaviors for academic success. As medical education continues to evolve, pedagogical approaches that prioritize student engagement, such as the WSLA, may be favored to foster a deeper and more enduring understanding of essential knowledge in the health sciences. Future research should delve deeper into the factors that influence sustained student engagement and explore additional strategies to increase the effectiveness of integrated curricula. Future research should delve deeper into the factors that influence sustained student engagement and, more specifically, the mechanisms that underlie the positive effects of WSLA. This will open new avenues for increasing the effectiveness of integrated curricula.

### Electronic supplementary material

Below is the link to the electronic supplementary material.

Supplementary Material 1

## Data Availability

The datasets generated and/or analysed during the current study are available from the corresponding author on reasonable request.
